# Correlation of Circulating MMP-9 with White Blood Cell Count in Humans: Effect of Smoking

**DOI:** 10.1371/journal.pone.0066277

**Published:** 2013-06-25

**Authors:** Soren Snitker, Keming Xie, Kathleen A. Ryan, Daozhan Yu, Alan R. Shuldiner, Braxton D. Mitchell, Da-Wei Gong

**Affiliations:** 1 Division of Endocrinology, Diabetes and Nutrition, Department of Medicine, University of Maryland School of Medicine, Baltimore, Maryland, United States of America; 2 Department of Pathophysiology, Medical College of Soochow University, Suzhou, China; 3 Geriatrics Research and Education Clinical Center, Baltimore Veterans Affairs Medical Center, Baltimore, Maryland, United States of America; Sapienza University of Rome, Italy

## Abstract

**Background:**

Matrix metalloproteinase-9 (MMP-9) is an emerging biomarker for several disease conditions, where white blood cell (WBC) count is also elevated. In this study, we examined the relationship between MMP-9 and WBC levels in apparently healthy smoking and non-smoking human subjects.

**Methods:**

We conducted a cross-sectional study to assess the relationship of serum MMP-9 with WBC in 383 men and 356 women. Next, we divided the male population (women do not smoke in this population) into three groups: never (n = 243), current (n = 76) and former (n = 64) smokers and compared the group differences in MMP-9 and WBC levels and their correlations within each group.

**Results:**

Circulating MMP-9 and WBC count are significantly correlated in men (R^2^ = 0.13, p<0.001) and women (R^2^ = 0.19, p<0.001). After stratification by smoking status, MMP-9 level was significantly higher in current smokers (mean ± SE; 663.3±43.4 ng/ml), compared to never (529.7±20.6) and former smokers (568±39.3). WBC count was changed in a similar pattern. Meanwhile, the relationship became stronger in current smokers with increased correlation coefficient of r = 0.45 or R^2^ = 0.21 (p<0.001) and steeper slope of ß = 1.16±0.30 (p<0.001) in current smokers, compared to r = 0.26 or R^2^ = 0.07 (p<0.001) and ß = 0.34±0.10 (p<0.001) in never smokers.

**Conclusions:**

WBC count accounts for 13% and 19% of MMP-9 variance in men and women, respectively. In non-smoking men, WBC count accounts for 7% of MMP-9 variance, but in smoking subjects, it accounts for up to 21% of MMP-9 variance. Thus, we have discovered a previously unrecognized correlation between the circulating MMP-9 and WBC levels in humans.

## Introduction

Matrix metalloproteinases (MMPs) are a family of endopeptidases that degrade extracellular matrix proteins (ECMs), play a pivotal role in tissue remodeling, and participate in a variety of physiological and pathological processes [1],[2],[3],[4]. One of the members of the MMP family, MMP-9, is a gelatinase that has been implicated in the pathogenesis of atherosclerosis [5] and chronic obstructive pulmonary disease (COPD) [6], [7] in addition to tumor formation and metastasis [8],[9]. Accordingly, a number of studies have associated elevated serum levels of MMP-9 with many chronic inflammatory conditions including coronary artery disease (CAD) [10],[11],[12],[13],[14],[15],[16], COPD [17],[18],[19], arthritis [20],[21],[22] and metabolic syndrome [23],[24]. Thus, MMP-9 has emerged as a novel disease marker [25], [26], [27] as well as a therapeutic target [28],[29]. However, to make MMP-9 a clinically meaningful risk marker, the source of elevated MMP-9 levels in a variety of states must be better understood.

MMP-9 is broadly expressed in many tissues and types of cells, including lungs [30], [31], heart [32],[33], brain [34], neutrophils [35],[36], smooth muscle cells, endothelium [37] and cancer cell lines [38]. While the source(s) of circulating MMP-9 levels has not been unequivocally established, both *in vitro* and *in vivo* studies indicate that neutrophils can be such a source. *In vitro*, leukocyte MMP-9 gene expression and protein release are stimulated by inflammatory mediators such as phorbol 12-myristate 13-acetate (PMA), tumor necrosis factor-α (TNFα) and bacterial lipopolysaccharides (LPS) [39],[40],[41],[42]. In healthy human subjects given LPS intravenously, plasma MMP-9 levels rise rapidly in a pattern that matches its release of isolated neutrophils *in vitro* in the same subjects, suggesting that neutrophils are a likely source of MMP-9 in an overt and acute inflammatory condition [43]. In an attempt to determine the source of circulating MMP-9 in a non-infectious, inflammatory state, Jonsson et al. [44] fractionated blood cells from patients with CAD and from healthy controls into peripheral blood mononuclear cells (PBMCs) and neutrophils, and found that the dominant source of MMP-9 is neutrophils and that neutrophils from CAD patients secreted more MMP-9 than those from the controls *in vitro*. However, no statistical difference in circulating MMP-9 levels was detected between the CAD patients and controls.

Intriguingly, in subclinical inflammatory conditions where MMP-9 is elevated, such as CAD, COPD and metabolic syndrome, white blood cell count (WBC) is frequently elevated [23], [24], [45], [46], [47], [48], although MMP-9 and WBC are usually studied separately. The joint association of these factors with inflammatory diseases, along with the *in vitro* mechanistic studies [39],[40],[41],[42],[44], raises the possibility that leukocytes could be a major source of the circulating MMP-9 in humans, especially in an inflammatory condition. Smoking is a common cause of non-infectious, subclinical inflammation. We thus hypothesized that (1) MMP-9 will be correlated with WBC at the population level, (2) MMP-9 levels will be higher in smokers than in never-smokers, and (3) MMP-9 levels for a given WBC level will be higher in current smokers than in never and former smokers. We tested these hypotheses in a well-defined, apparently healthy population.

## Methods

### Study subjects

Subjects included in this study were relatively healthy adults aged 20 years or older from the Amish population in Lancaster County, Pennsylvania, who participated in the Heredity and Phenotype Intervention (HAPI) Heart Study [49],[50]. The primary goal of this study was to identify genes that interact with environmental exposures to modify risk factors for cardiovascular disease. Study-wide exclusion criteria included: 1) age <20 years, 2) currently pregnant or postpartum <6 months, 3) blood pressure at the time of screening >180/105 mm Hg, and 4) coexisting malignancy.

All subjects provided their written informed consent to participate in the study and the Institutional Review Board of University of Maryland for Human Research approved the study.

### Examination of subjects and laboratory methods

All study participants underwent a physical examination during their visit to the Amish Research Clinic in Strasburg, PA. Subjects were withdrawn from all medications, vitamins and supplements for seven days prior to their initial assessment. Height and weight were measured using a stadiometer and calibrated scale with shoes removed and in light clothing, and body mass index (BMI) (kg/m^2^) was computed. Blood pressure was measured in triplicate in the sitting position after the subject had been sitting quietly for 5 min by use of a standard sphygmomanometer, and the average of the measurements was calculated. Participants reported their smoking habits, including whether they currently or ever smoked, the amount they smoked, and the years they started and, if applicable, quit smoking, from which the duration and lifetime dose of cigarette smoking could be calculated. Blood samples were drawn in the fasting state and placed at 4°C, processed within 30 to 60 minutes, and frozen at −80°C until laboratory assay. Total cholesterol, low and high density lipoprotein cholesterol (LDL-C and HDL-C), alanine aminotransferase (ALT), hemoglobin, red and white blood cell counts, and HCT were assayed by Quest Diagnostics (Lancaster, PA). Glucose was measured with a YSI glucose analyzer using the glucose oxidase method at the University of Maryland, General Clinical Research Center (Baltimore, MD). Insulin was measured by radioimmunoassay at Johns Hopkins Bayview Medical Center (Baltimore, MD). Serum concentrations of IL-1β were measured in triplicate and MMP-1 and MMP-9 concentrations in duplicate using an enzyme-linked immunosorbent assay (ELISA) (R&D Systems, Minneapolis, MN) by the University of Maryland Cytokine Biochemistry Core Laboratory. SAA was measured in duplicate by ELISA (Biosource, Camarillo, CA). The means of the replicate values were used for data analyses. The assay detection ranges were 0.781 to 50 pg/ml for IL-1β, 0.156 to 10 ng/ml for MMP-1, 31.2 to 2000 ng/ml for MMP-9, 5.0 to 600 ng/ml for SAA. Samples above the maximum detection values were diluted for measurement. The intra-assay coefficients of variation (CV) were 5.5%, 7.5%, 5.8% and 4.9% for IL-1β, MMP-1, MMP-9 and SAA, respectively. High-sensitive CRP, determined by End Point Nephelometry, was assayed by Quest Diagnostics with inter-assay CV of 4.8%.

### Statistical analysis

**Figure 1:**
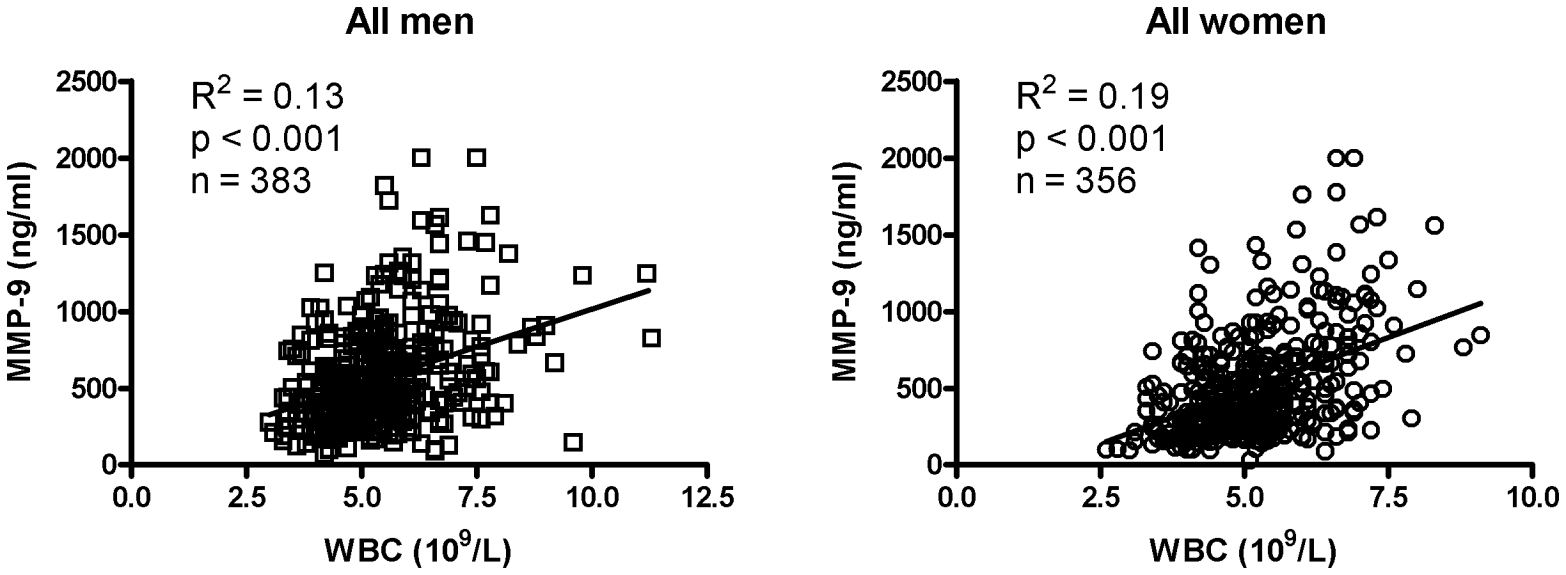
Correlation between MMP-9 and WBC in apparently healthy subjects. Linear regression analysis of the correlation between MMP-9 and WBC in men (n = 383) and women (n = 356).

**Figure 2:**
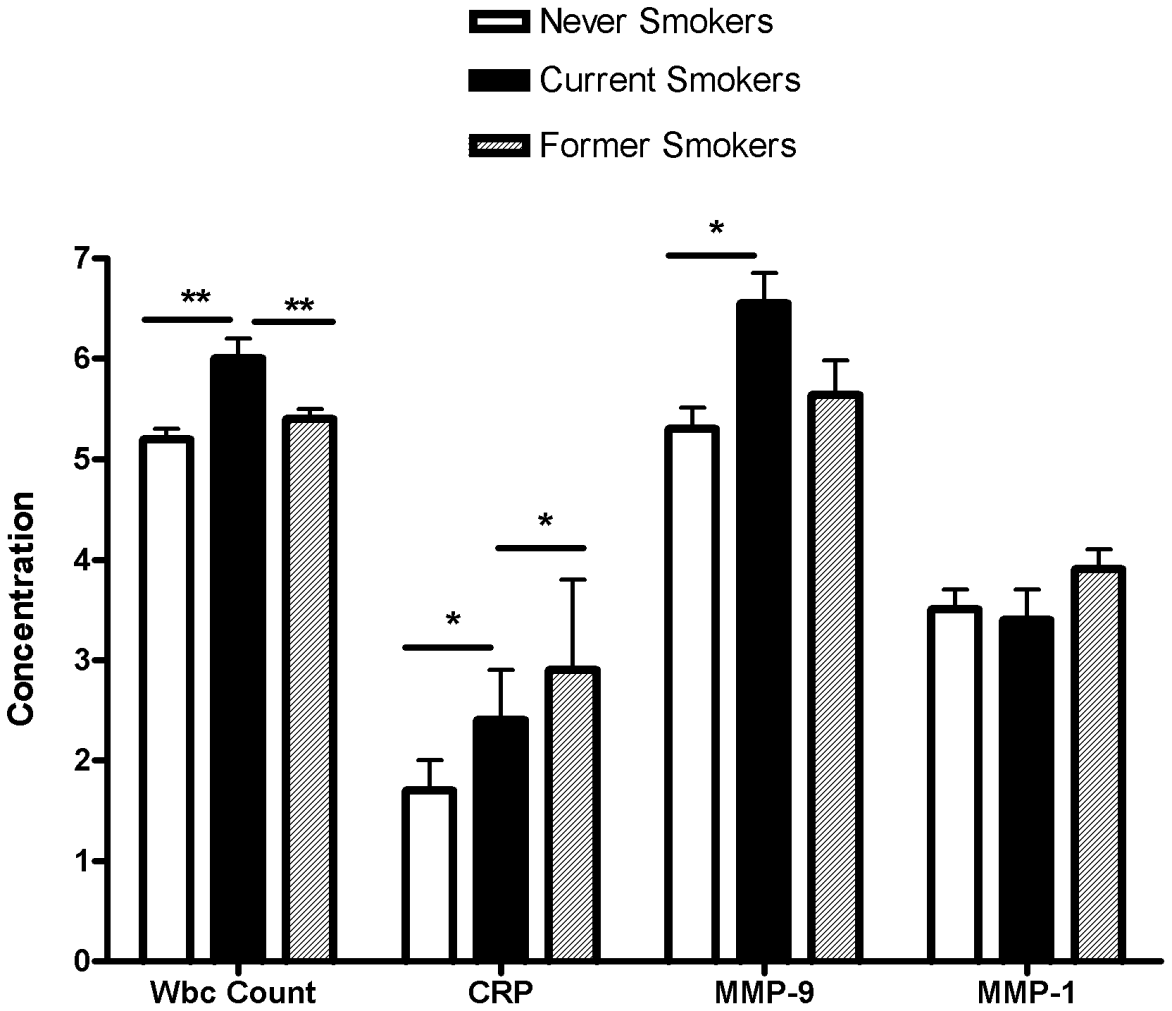
Plasma concentrations of inflammatory markers according to smoking status. White blood cell (WBC) count is expressed as (5×10^9^/L), C-reactive protein (CRP), MMP-9 and MMP-1 as ng/ml. logCRP and logMMP-9 were used for analyses. *: p<0.05; **: p<0.01.

Data are reported as mean ± standard error (SE) unless otherwise stated. Group differences and regression analyses were carried out in a variance component framework in which we incorporated relatedness among study participants as a random effect (polygenic component), as implemented in the SOLAR software program (version 4.07 Southwest Foundation for Biomedical Research, San Antonio, TX). We computed mean levels of WBC and multiple inflammatory markers (e.g. MMP-1, interleukin 1β (IL-1β), MMP-9, CRP, and SAA) by quartile of MMP-9 to assess the correlations of these variables with MMP-9. We then tested the correlations between MMP-9 and each of these factors using a regression-based approach with adjustment for age and body mass index (BMI). MMP-9 was natural logarithm transformed prior to analysis, and the transformed values were approximately normally distributed. Similarly, we logarithm transformed other non-normally distributed variables (e.g., CRP and MMP-1) prior to analysis. For Figs. 1 and 2, we employed Pearson's correlation analysis to calculate correlations between variables and ANOVA for comparison of cytokine mean differences between smokers and non-smokers (GraphPad Software; La Jolla, CA). We consider a p value<0.05 statistically significant.

## Results

### Association of MMP-9 with WBC count in men and women

Basic clinical characteristics and cytokine levels of the study subjects are summarized and stratified by gender in Table 1. Compared with women, men had higher values of HDL, ALT, diastolic blood pressure (DBP), RBC count, MMP-9 and CRP levels, whereas they had lower values of BMI, platelet count and SAA. To explore the correlation between MMP-9 and WBC count, we first examined the association in the whole population regardless of smoking status. Since MMP-9 levels differed between men and women, we conducted analyses separately for men and women. We first compared select anthropometric and biochemical markers according to MMP-9 quartile and determined the *P* for trend. We adjusted for age and BMI, as these variables influence many laboratory measurements. Tables 2 and 3 show the results for men and women, respectively. In men, (Table 2), MMP-9 level is strongly associated with WBC count and less significantly with RBC count, MMP-1 and SAA levels, but not with IL-1ß and CRP levels. In women (Table 3), MMP-9 level is associated with WBC, MMP-1, IL-1ß and CRP, but not with RBC. The fact that MMP-9 levels are correlated with MMP1, CRP and RBC opens a possibility of co-regulation among these parameters. To exclude the possible confounding effect, the association of MMP-9 with WBC was further examined after adjustment for these factors individually and remained strong (Tables 2 and 3).

**Table 1:** Basic Clinical Characteristics and Cytokine Levels of Study Participants by Gender.

	Women (n = 356)	Men (n = 383)	p-value**
AGE (years)	45.0±0.7 (20–76)	41.5±0.7 (20–77)	**0.0007**
BMI (kg/m^2^)	27.7±0.3 (17.3–46.8)	25.5±0.2 (18.4–38.0)	**4.4E-09**
Cholesterol (mg/dl)	217.1±2.7 (103–413)	204.7±2.2 (75–379)	0.06
HDL	59.6±0.8 (27–116)	53.1±0.7 (24–100)	**4.6E-11**
LDL	143.0±2.5 (28–330)	138.8±2.1 (17–290)	0.54
Triglycerides* (mg/dl)	72.4±2.3 (9.9–255)	63.8±1.9 (11–298)	0.31
Glucose*	86.1±0.6 (48–173)	86.7±(49–209)	0.45
Insulin*	9.6±0.3 (0.5–45.4)	8.5±0.2 (0.4–25.2)	0.03
ALT (U/L)*	16.7±0.3 (6–44)	20.7±0.4 (8–71)	**1.9E-21**
SBP (mm Hg)	121.2±0.9 (83–183)	121.1±0.6 (93–163)	0.15
DBP (mm Hg)	75.8±0.4 (58–107)	77.8±0.5 (41–108)	**2.3E-05**
WBC (10^3^/µl)	5.2±0.1 (2.6–9.1)	5.4±0.1 (3.0–11.3)	**0.05**
RBC(10^12^/L)*	4.26±0.02 (3.53–5.11)	4.78±0.02 (3.82–5.80)	**1.0E-92**
Platelet (10^9^/L) *	242.2±2.8 (94–570)	228.9±2.6 (81–551)	**0.004**
MMP-1 (ng/ml)*	3.9±0.1 (0.2–10.0)	3.6±0.1 (0.2–10.0)	0.18
IL-1β (ng/ml)*	4.6±0.6 (0.8–50.0)	4.1±0.5 (0.8–50.0)	0.89
MMP-9 (ng/ml)*	512.8±18.1 (31–2000)	562.7±17.1 (68–2000)	**0.008**
CRP (ng/ml)*	1.9±0.1 (0.2–23.0)	2.1±0.3 (0.2–78.6)	**0.05**
SAA (µg/ml)*	25.9±2.4 (2–298)	13.0±1.0 (0.4–107)	**4.6E-11**

Values are Mean ± SE (range).

* : Log-transformed for analysis;

** : Except for age, all p values are adjusted for age; Bold for p≤0.05.

**Table 2:** Association of MMP-9 Quartiles with Selected Clinical Characteristics in Men.

	Quartile 1	Quartile 2	Quartile 3	Quartile 4		P for trend	P for trend
	MMP-9:	MMP-9:	MMP-9:	MMP-9:	P for trend	Adjusted for	Adjusted for
	68–316	319–477	485–726	727–2000	Adjusted for	age, BMI, &	age, BMI, &
	(n = 95)	(n = 96)	(n = 96)	(n = 96)	age, BMI	MMP-1	RBC
AGE (years)	40.8±1.4	40.1±1.3	41.1±1.4	44.1±1.3	NA	NA	NA
BMI (kg/m^2^)	24.8±0.3	25.5±0.4	25.5±0.3	26.2±0.3	NA	NA	NA
SBP (mm Hg)	122±1	121±1	121±1	120±1	0.07	0.06	**0.04**
DBP (mm Hg)	78±1	77±1	79±1	78±1	0.61	0.60	0.37
WBC (10^9^/L)	5.0±0.1	5.1±0.1	5.4±0.1	6.0±0.2	**1.3E-09**	**2.9E-09**	**2.2E-08**
RBC(10^12^/L)*	4.73±0.03	4.74±0.03	4.83±0.03	4.82±0.03	**0.003**	**0.003**	NA
MMP-1 (ng/ml)	3.2±0.2	3.6±0.2	3.5±0.2	4.0±0.3	**0.02**	NA	**0.02**
IL-1ß (ng/ml)	2.9±0.8	5.2±1.2	4.4±1.1	4.0±1.0	0.48	0.40	0.42
CRP (ng/ml)	2.2±0.8	1.5±0.3	1.6±0.3	3.3±0.9	0.07	0.09	0.07
SAA (µg/ml)	11.6±1.8	13.3±2.1	11.6±1.8	17.2±2.5	**0.05**	0.07	**0.04**

**Table 3:** Association of MMP-9 Quartiles with Selected Clinical Characteristics in Women.

	Quartile 1	Quartile 2	Quartile 3	Quartile 4		P for trend	P for trend
	MMP-9:	MMP-9:	MMP-9:	MMP-9:	P for trend	Adjusted for	Adjusted for
	31–263	263–413	413–677	683–2000	Adjusted for	age, BMI, &	age, BMI, &
	(n = 89)	(n = 89)	(n = 89)	(n = 89)	age, BMI	CRP	MMP-1
AGE (years)	47.5±1.6	46.2±1.4	43.5±1.4	43.0±1.4	NA	NA	NA
BMI (kg/m^2^)	27.1±0.6	28.4±0.6	27.2±0.5	28.2±0.5	NA	NA	NA
SBP (mm Hg)	124±2	121±2	118±2	122±2	0.47	0.53	0.32
DBP (mm Hg)	77±1	75±1	76±1	75±1	0.16	0.23	0.20
WBC (10^9^/L)	4.7±0.1	5.0±0.1	5.2±0.1	5.9±0.1	**1.6E-14**	**4.3E-12**	**1.6E-12**
RBC (10^12^/L)*	4.26±0.03	4.26±0.03	4.24±0.03	4.27±0.03	0.34	0.37	0.60
MMP-1 (ng/ml)	3.6±0.3	3.6±0.2	3.5±0.2	4.9±0.3	**1.2E-05**	**2.2E-04**	NA
IL-1ß (ng/ml)	2.5±0.6	4.2±1.2	6.6±1.4	5.3±1.3	**0.008**	**0.004**	**0.006**
CRP (ng/ml)	1.5±0.2	1.7±0.2	1.5±0.2	2.7±0.4	**1.1E-04**	NA	**0.002**
SAA (ug/ml)	19.2±1.7	24.9±2.7	31.9±8.3	37.3±11.3	0.12	0.40	0.22

Next, we examined the association of WBC count by quartiles vs. the same set of parameters in men and women. Tables 4 and 5 show that WBC count is associated with MMP-9 and CRP levels in both sexes, but with RBC count only in men and MMP-1 only in women. After adjustment for the factors individually, WBC count still hold a strong association with MMP-9. Moreover, the association remains strong regardless of the adjustment for CRP in the above analyses. Thus, the association of MMP-9 with WBC is independent of RBC, MMP-1, CRP, and other biochemistry parameters (data not shown). Fig. 1 recapitulates the correlation of MMP-9 with WBC in men (r = 0.33, p<0.001) and women (r = 0.42, p<0.001) in scatter plot.

**Table 4:** Association of WBC Quartiles with Selected Clinical Characteristics in Men.

	Quartile 1	Quartile 2	Quartile 3	Quartile 4		P for trend	P for trend
	WBC:	WBC:	WBC:	WBC:	P for trend	Adjusted for	Adjusted for
	3.0–4.5	4.6–5.1	5.2–5.9	6.0–11.3	Adjusted for	age, BMI, &	age, BMI, &
	(n = 93)	(n = 85)	(n = 104)	(n = 101)	age, BMI	CRP	RBC
AGE (years)	39.9±1.4	40.6±1.4	39.6±1.2	45.8±1.4	NA	NA	NA
BMI (kg/m^2^)	24.8±0.3	25.3±0.3	25.6±0.3	26.3±0.3	NA	NA	NA
SBP (mm Hg)	120±1	121±1	121±1	122±1	0.13	0.18	0.21
DBP (mm Hg)	77±1	77±1	79±1	78±1	0.50	0.42	0.19
RBC(10^12^/L)*	4.70±0.03	4.72±0.03	4.86±0.03	4.82±0.03	**1.0E-05**	**5.7E-06**	NA
MMP-1 (ng/ml)	3.1±0.2	3.6±0.3	3.5±0.2	4.1±0.3	0.09	0.24	0.07
MMP-9 (ng/ml)	418±24	475±23	597±33	735±41	**1.1E-09**	**6.6E-09**	**1.2E-08**
IL-1ß (ng/ml)	5.0±1.2	5.5±1.4	4.0±0.8	2.2±0.5	0.41	0.42	0.47
CRP (ng/ml)	1.4±0.3	1.4±0.3	2.1±0.8	3.4±0.8	**7.5E-08**	NA	**3.9E-08**
SAA (µg/ml)	10.6±1.7	12.5±1.8	11.9±2.0	16.8±2.4	**0.02**	0.35	**0.006**

**Table 5:** Association of WBC Quartiles with Selected Clinical Characteristics in Women.

	Quartile 1	Quartile 2	Quartile 3	Quartile 4		P for trend	P for trend
	WBC:	WBC:	WBC:	WBC:	P for trend	Adjusted for	Adjusted for
	2.6–4.3	4.4–5.0	5.1–5.8	5.9–9.1	Adjusted for	age, BMI, &	age, BMI, &
	(n = 78)	(n = 85)	(n = 101)	(n = 91)	age, BMI	CRP	MMP-1
AGE (years)	47.0±1.7	45.8±1.5	43.9±1.3	44.1±1.4	NA	NA	NA
BMI (kg/m^2^)	26.6±0.6	26.6±0.5	28.1±0.5	29.2±0.6	NA	NA	NA
SBP (mm Hg)	123±2	122±2	119±2	121±2	0.84	0.96	0.67
DBP (mm Hg)	77±1	77±1	75±1	75±1	0.08	0.14	0.10
RBC(10^12^/L)*	4.22±0.03	4.19±0.03	4.30±0.03	4.32±0.03	0.07	**0.05**	**0.02**
MMP-1 (ng/ml)	3.3±0.2	4.1±0.3	3.5±0.2	4.8±0.3	**1.8E-04**	**0.005**	NA
MMP-9 (ng/ml)	380±29	419±22	475±28	758±46	**1.1E-14**	**3.1E-12**	**9.7E-13**
IL-1ß (ng/ml)	3.7±1.1	4.9±1.2	4.8±1.2	5.0±1.3	0.81	0.98	0.84
CRP (ng/ml)	1.2±0.1	1.5±0.2	1.6±0.2	3.1±0.4	**9.0E-08**	NA	**2.3E-06**
SAA (µg/ml)	21.3±2.6	32.5±6.3	19.1±2.7	30.8±6.5	0.09	0.89	0.18

### Elevation of MMP-9 and WBC in current smokers

The comparison of MMP-9 levels between smokers and non-smokers was restricted to Amish men, since Amish women do not smoke. To examine whether cigarette smoking influences MMP-9 and WBC levels, we stratified men into three groups: never (n = 243), current (n = 76) and former (n = 64) smokers and compared the changes of MMP-9 and WBC, along with CRP and MMP-1, among the groups. Fig. 2 shows that MMP-9 was 25.2% higher in current than never smokers (current vs. never; 663.3+43.4 vs. 529.7+20.6 ng/ml, p<0.01) and its level was reduced by 14% to 568.0+39.3 ng/ml in former smokers. Likewise, WBC count in current smokers was 17.7% higher than never smokers (6.1+0.2 vs. 5.2+0.1×10^9^/L, p<0.001). An 8% decrease of WBC was found in former smokers (5.5+0.2×10^9^/L), but the level remained significantly higher than that of never smokers. For comparison, the CRP level was higher in current smokers by 41.7% (p<0.01 vs. never smokers) and further increased by 39.8% in former smokers but with no statistical significance vs. never and current smokers. In contrast, MMP-1 levels did not differ among the three groups.

### Enhanced Correlation between MMP-9 and WBC in current smokers

**Figure 3:**
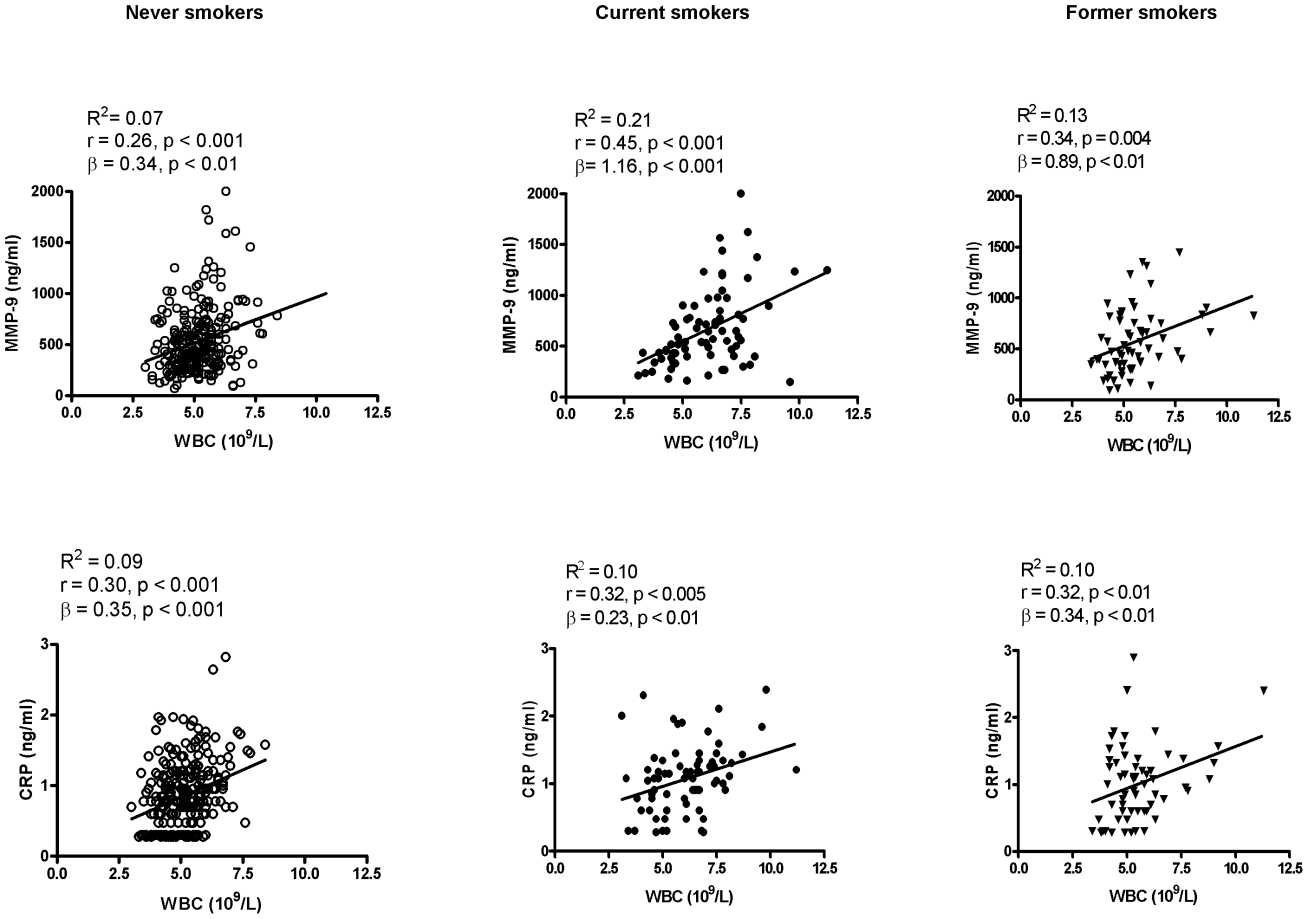
Correlations of MMP-9 and CRP with WBC. Linear regression analysis of the correlation of WBC with MMP-9 (upper panel) and CRP (lower panel) in never (n = 243), current (n = 76) and former smokers (n = 64).

A similar regulation pattern of MMP-9 and WBC by smoking status suggests a possible co-regulation between the two variables (Fig. 2). We next performed linear regression analysis to assess whether the correlation of MMP-9 vs. WBC count varied by smoking status, in comparison with that of CRP vs. WBC count. As shown in Fig. 3, a weak correlation was observed between MMP-9 and WBC count in never smokers (R^2^ = 0.07 or r = 0.26, p<0.0001; ß = 0.34, p<0.001), and the relationship became stronger in current smokers (R^2^ = 0.21 or r = 0.45, p<0.001; ß = 1.16, p<0.001) and remained significant in former smokers (R^2^ = 0.13 or r = 0.34, p<0.01; ß = 0.89, p<0.001). As a result, WBC account for 7% of MMP-9 variants in never smokers but for 21% in current smokers. Further ß interaction analysis indicates that the slope of MMP-9 and WBC relationship is significantly steeper (p<0.001) in current and former smokers than never smokers. For comparison, although CRP is correlated with WBC count, its relationship does not vary appreciably with smoking status in terms of the correlation coefficient (R^2^ = ∼0.1) and slope (ß = 0.23–0.35).

## Discussion

In this study, we examined the MMP-9 and WBC relationship in a relatively healthy population. We have found that circulating MMP-9 levels are positively correlated with WBC in men and women. Moreover, both markers are elevated and the relationship improves in respect of the correlation coefficient and ß value in smoking subjects. The finding is significant in that it provides epidemiological evidence supporting that WBC count is a likely source of circulating MMP-9 *in vivo* and that cigarette smoking is a notable influencing factor for MMP-9 as well as WBC elevation. Previous studies have suggested that neutrophils can be responsible for the circulating MMP-9 during a bacterial inflammatory challenge in humans [43] but evidence for neutrophils as a source of MMP-9 in non-infectious conditions is lacking. In the present study, we have first demonstrated a positive correlation of MMP-9 with WBC count in both men and women (Tables 2–5, and Fig. 1), which is independent of other inflammatory cytokines (e.g., CRP, MMP-1, SAA and IL-1ß), demonstrating a positive association in an apparently healthy population. Then, we stratified men depending on smoking status, a known condition of non-bacterial, subclinical inflammation, and found that both MMP-9 and WBC levels were elevated in current smokers and reduced in former smokers. Thus, current smoking status is associated with elevated MMP-9 levels. Meanwhile, the correlation between WBCs and MMP-9 levels was stronger in smokers than in non-smokers. Although cause and effect cannot be determined from cross-sectional studies, it is logical to reason that the WBC count increase is a likely cause of MMP-9 elevation, because MMP-9 is derived from WBCs, but not *vice versa*. Thus, the stronger relationship suggests that WBC count accounts for a larger proportion of the variation in MMP-9 levels in smokers than in non-smokers, or possibly that WBCs secrete more MMP-9 under an ongoing smoking condition.

Our finding of the previously unrecognized *in vivo* relationship of MMP-9 levels with WBC count has the following biological and clinical implications: 1) In non-smoking relatively healthy subjects, MMP-9 may be spontaneously released from WBC, which accounts for up to 7 and 19% MMP-9 variation in men and women, respectively (Fig. 2 and Fig. 3); 2) Under a non-infectious inflammatory state such as smoking, WBCs can account up for 21% of MMP-9 variation and may release more MMP-9, as suggested by the stronger correlation in smokers vs non-smokers. Elevation of both circulating MMP-9 and WBC count have been considered risk factors or biomarkers for cardiovascular disease [10],[11],[12],[13],[14],[15],[16],[51],[52],[53], COPD [17],[18],[19], arthritis [20],[21],[22] and metabolic syndrome [23],[24], all of which are associated with subclinical inflammation and are promoted by smoking. The close relationship of MMP-9 with WBC count in smoking status provides an explanation for the overlap of these two biomarkers for those diseases; 3) As MMP-9 is an emerging biomarker for many inflammation-related diseases, caution should be exercised that WBC count can be a confounding factor.

Nevertheless, the functional consequence of MMP-9 elevation during smoking requires further study. MMP-9 activity is suppressed by the endogenous tissue inhibitors of metalloproteinase 1 (TIMP1) [54],[55] and is a result of the balance between MMP-9 and TIMP-1 [56],[57]. As we did not measure TIMP-1 concentration or MMP-9 activity in the study, whether the elevated MMP-9 during smoking leads to increased MMP-9 activity is not known but deserves further study. It is also important to note that our finding of MMP-9 and WBC relationship is observational. Subsequent clinical study to fractionate blood cells and isolate neutrophils from smokers and none smokers is required to provide a mechanistic explanation for the relationship.

In summary, we have found a significant correlation of MMP-9 with WBC count in apparently healthy subjects. Moreover, the levels of MMP-9 and WBC and their relationship are augmented in smoking subjects. Together with published in vitro studies, our observations support the notion that WBCs are a source of circulating MMP-9, especially in the smoking-induced, subclinical, inflammatory state.
